# Controlled nanodot fabrication by rippling polycarbonate surface using an AFM diamond tip

**DOI:** 10.1186/1556-276X-9-372

**Published:** 2014-07-30

**Authors:** Yongda Yan, Yang Sun, Jiran Li, Zhenjiang Hu, Xuesen Zhao

**Affiliations:** 1 The State Key Laboratory of Robotics and Systems, Robotics Institute, Harbin Institute of Technology, Harbin, Heilongjiang 150008, P.R. China; 2 Center for Precision Engineering, Harbin Institute of Technology, Harbin, Heilongjiang 150001, People’s Republic of China; 3 Harbin Institute of Technology, P.O. Box 413, Harbin, Heilongjiang 150001, China

**Keywords:** Atomic force microscope (AFM), Polycarbonate (PC), Scratching, Ripples

## Abstract

The single scratching test of polymer polycarbonate (PC) sample surface using an atomic force microscope (AFM) diamond tip for fabricating ripple patterns has been studied with the focus on the evaluation of the effect of the tip scratching angle on the pattern formation. The experimental results indicated that the different oriented ripples can be easily machined by controlling the scratching angles of the AFM. And, the effects of the normal load and the feed on the ripples formation and their periods were also studied. Based on the ripple pattern formation, we firstly proposed a two-step scratching method to fabricate controllable and oriented complex three-dimensional (3D) nanodot arrays. These typical ripple formations can be described via a stick-slip and crack formation process.

## Background

Nowadays, organic polymers have replaced many traditional engineering materials because of their superior performance and low cost [1]. The polymer-based nanoscale structures are used in a wide range of important applications, such as bioengineering, grating sensors, binary optics, and so on [2,3]. One of the essential technologies used to fabricate nanoscale structures is atomic force microscopy (AFM), which is a tip-based nanomechanical machining method that possesses the advantages of precise spatial resolution, *in situ* imaging, and other unique features, including the inexpensive device, relatively easy control and operation [4]. Especially, the AFM-based friction-induced nanomechanical method, which belongs to one of the AFM-based nanofabrication methods, is looked on as a new way for forming complex nanostructures [5,6].

Ripple patterns can exist over a range of length scales including macroscopic linear ripples on sea and desert sands created by wind [7], microsized ripples on surfaces of metal substrates produced by ion sputtering [8], and nanoscale ripples on the surfaces of thermoplastic polymers obtained by an atomic force microscope (AFM) tip’s reciprocal scanning [9]. In particular, it has been found that ripples can be formed on polymer surfaces by single scanning with an AFM tip. Acunto *et al*. [10,11] reported that ripple patterns could be formed with a small applied load and single scanning on the surfaces of solvent-containing polyethylene terephthalate (PET) films. Gnecco *et al*. [12] reported that linear ripples with the period of 100 to several hundreds of nanometers can be produced by a heated AFM tip on the surfaces of polycarbonate (PC), poly (methyl methacrylate) (PMMA), and PSul films, and the ripples could also be obtained with circular scanning. The main mechanisms for the tip-induced ripple formation including Schallamach waves, stick-slip, and fracture-based deformation [9,13,14] have been proposed. The Schallamach waves are reviewed as the inability of the rubber surface under high shear forces [9]. The stick-slip mechanism is the competition between the tangential force and the critical tangential force [13]. And, the fracture-based deformation is perceived as the existence of the cracks in the deformed materials [14]. All of the mechanisms are just the proposed model. They cannot be clearly conformed and came to an agreement for explaining the ripples’ formation. So, the mechanism for the process of such ripple formation is still controversial.

As mentioned above, just simple ripple-based structures had been formed by AFM tip’s scanning. And, for the novel friction-induced mechanical nanofabrication method, only the protrusive nanostructures including nanodots, nanolines, surface mesas, and nanowards have been produced by the mechanical interaction on the material surface. Until now, complex, ordered nanostructures on polymer surfaces using the friction-induced direct nanofabrication method are not reported [5,6]. In previous work, we produced nanoscale ripples by scratching a PC surface with an AFM tip with a hard cantilever once [15]. Such ripple patterns were actually quasi-sinusoidal structures that could be considered as typical three-dimensional (3D) nanostructures. To date, the formation of more complex polymer nanostructures by AFM scanning has not been reported.

Therefore, in the present paper, we use an AFM diamond tip with different scanning angles to trace a traditional zigzag pattern onto PC surfaces to study the effects of different scanning parameters including normal load and feed on the period of the resulting ripples. Based on these results, a novel two-step scanning method is then developed to realize controlled and oriented complex 3D nanodot arrays on PC surfaces. This permanent ripple structure appears to be caused by a stick-slip and crack formation process.

## Methods

**Figure 1:**
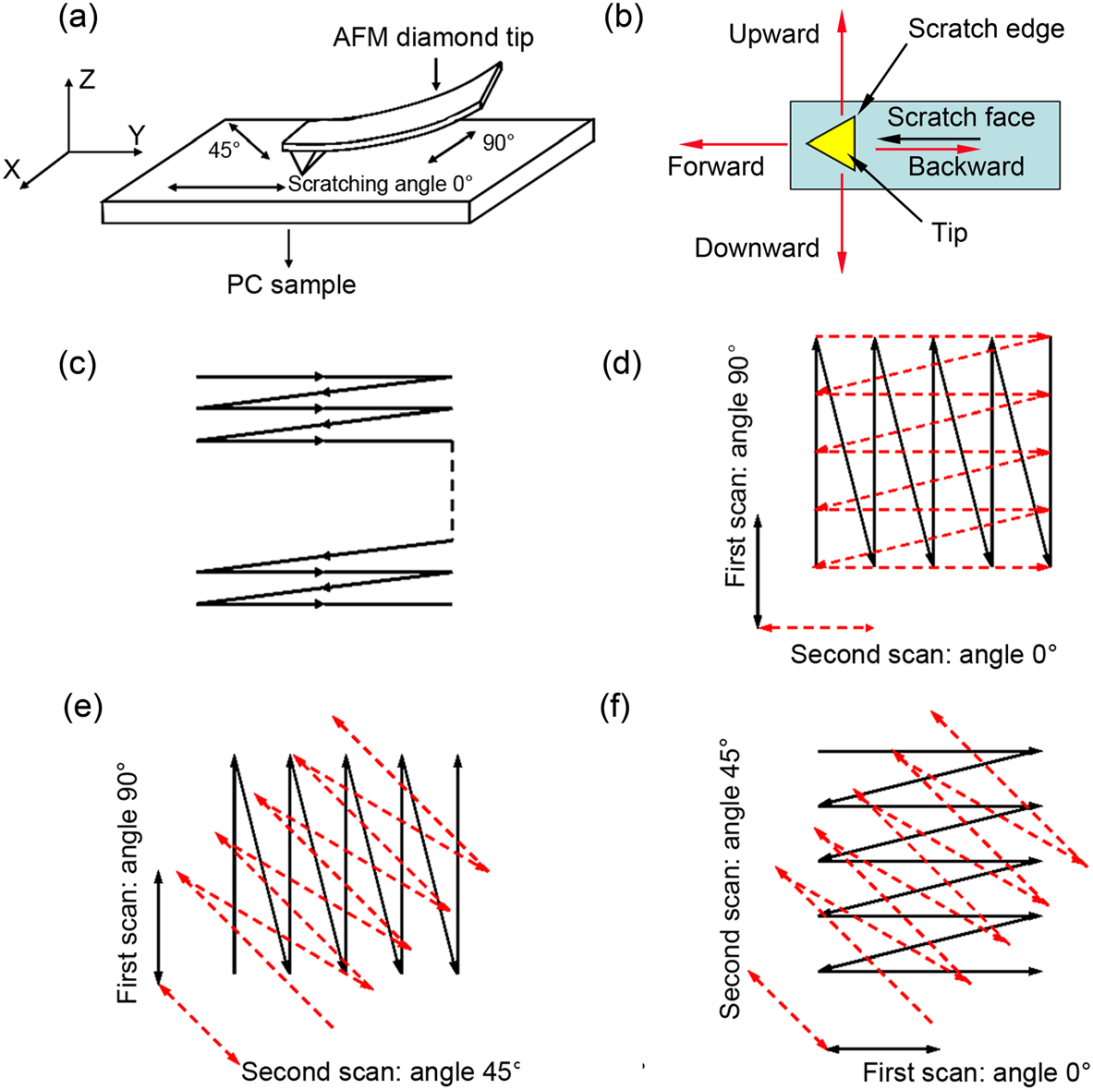
**Schematic of the nanobundles machining process. (a)** Schematic diagram showing the AFM scratching parameters and **(b)** the diamond tip, **(c)** zigzag trace of the AFM tip, and **(d) (e) (f)** a two-step method involving two consecutive tip scans with different scratching angles.

Injection-molded PC sample purchased from Yanqiao Engineering Plastics Co. Ltd. (Shanghai, China) was used as the sample. All experiments were carried out using an AFM (Dimension Icon, Bruker Company, Karlsruhe, Germany). A diamond tip (PDNISP, Veeco Company, Plainview, NY, USA) with a calibrated normal spring constant (K) of 202 N/m was used in contact mode to do all nanofabrication operations, and a silicon tip (RTESP, Veeco Company, Plainview, NY, USA) was used in tapping mode to obtain AFM images. The diamond tip is a three-sided pyramidal diamond tip (Figure 1b) with a radius *R* of 85 nm evaluated by the blind reconstruction method [16]. The PeakForce Quantitative NanoMechanics (QNM) microscopy was used to measure the modulus of material properties. The silicon tip (TAP525) with a normal spring constant (K) of 200 N/m was used to do the QNM test.A schematic diagram of the scratching test and the diamond tip are presented in Figure 1a,b, respectively. The front angle, back angle, and side angle are 55 ± 2°, 35 ± 2°, and 51 ± 2° for the tip. The fast scratching directions parallel at an angle of 45° and perpendicular to the long axis of the cantilever were named scratching angles 0°, 45°, and 90°, respectively. When scratching using the angle 0°, the tip scratch face and scratch edge are all perpendicular to the scratching direction. And, the cantilever tends to bend downward or upward under this situation; when scratching using the angle 90°, the tip scratch face and scratch edge are titled with an inclination angle with the scratching direction. And, the cantilever tends to twist under this situation; when scratching using the angle 45°, only the tip scratch face is titled with an inclination angle with the scratching direction. And, the cantilever tends to twist and bend simultaneously. Figure 1c shows the zigzag tip trace in the X-Y plane performed by the AFM system itself. Using the above three scratching angles, the tip scratched a zigzag trace into the sample surface in a given area. In view of this, a new two-step scratching method by combining two different scratching angles was proposed. Figure 1d,e,f shows the traces obtained by combining the scratching angles of 90° and 0°, 90° and 45°, and 0° and 45°, respectively. The solid black and dashed red lines represent the first and second traces, respectively. The intersectional areas shown in these images were the areas of the fabricated surfaces.

## Results and discussion

### Effect of scratching angle on ripple formation

**Figure 2:**
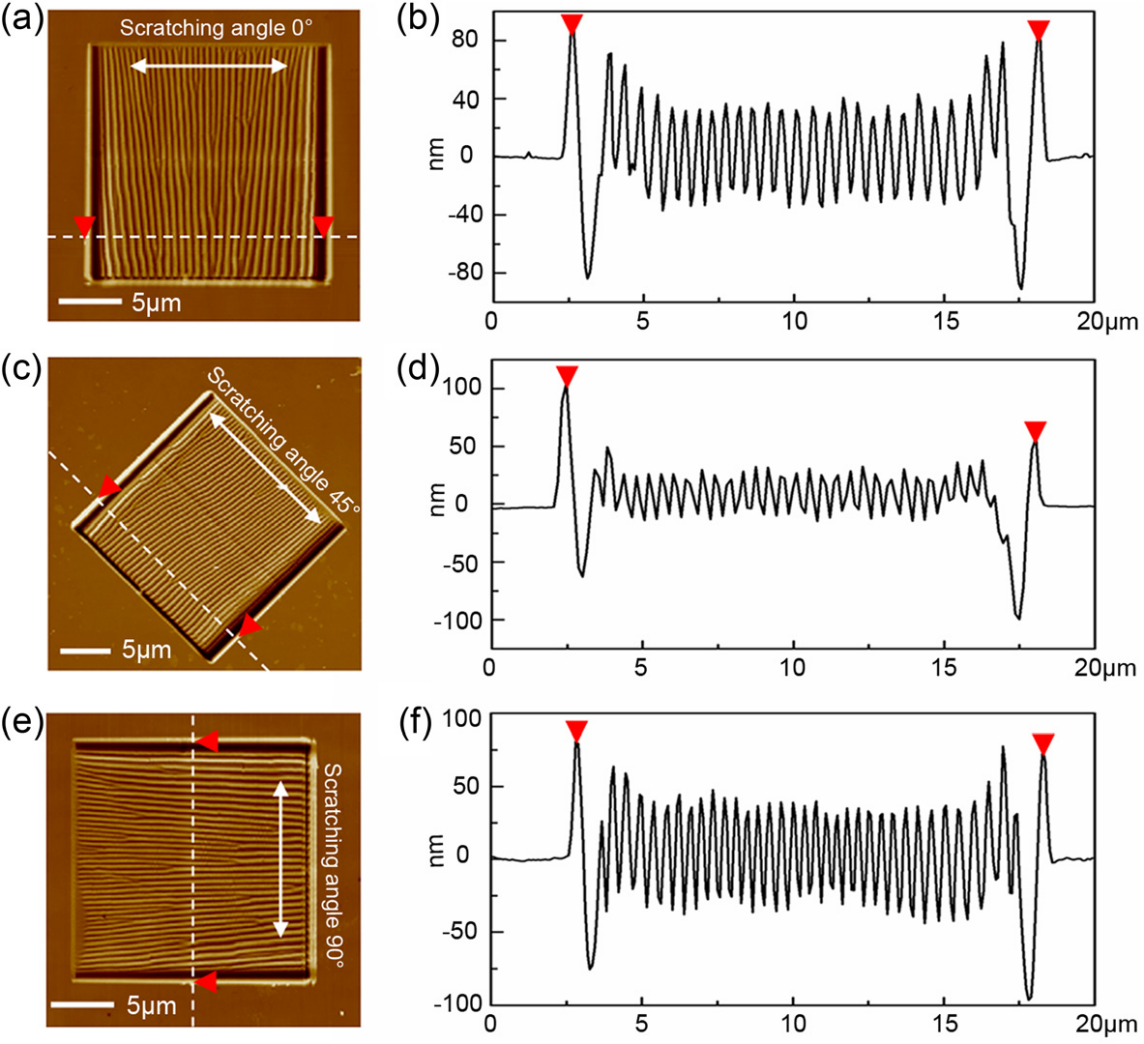
**The morphologies and cross-sections of the ripples.** The corresponding scratching angles are 0° **(a) (b)**, 45° **(c) (d)**, and 90° **(e) (f)**.

Scratching angles of 0°, 45°, and 90° were used to scratch PC surfaces with zigzag traces of the AFM tip. The machined structures and corresponding cross-sections are shown in Figure 2, with a scanning area of 15 μm × 15 μm, scan rate of 1 Hz, feed of 20 nm, and normal load of several micronewtons. The scratching velocity is 30 μm/s. Typical ripple patterns perpendicular to the scratching direction are formed on the PC surface for each scratching angle. Analysis of the section revealed that the ripple patterns are similar to sine-wave structures with a period of several hundred nanometers. In addition, some removed materials are all accumulated at the edge of the scanned area in the feeding direction for the three scratching angles. The reason for the accumulated materials may be due to the small quality of the removed materials piled up on the borders during the successive scanning. Based on the above experimental results, it can be obtained that the different oriented ripples can be easily machined by modulating the scratching angle of the tip.

### Effect of the machining parameters on the ripple formation

**Figure 3:**
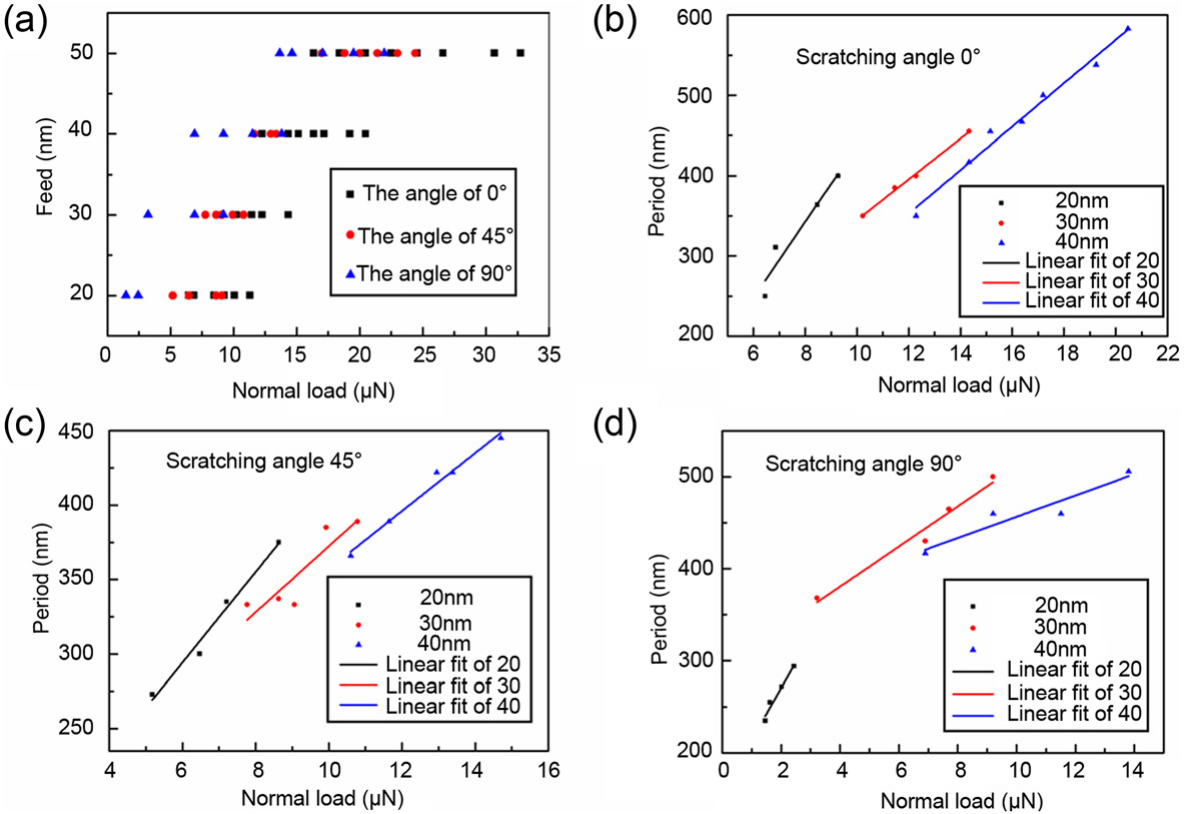
**The relationship between the feed, normal load and the ripple formation.** Effects of feed and normal load on **(a)** ripple formation and **(b)-(d)** the period of ripples for different scratching angles.

To obtain the machining parameters for ripple formation, feeds from 20 nm to 50 nm at 10-nm increments were investigated under different scratching angles by modulating the normal load. The obtained relationships between scratching parameters and ripple pattern formation are presented in Figure 3a. When the feed is 20 nm, the normal load for ripple formation ranges from 6.4 to 11.3 μN for scratching angle 0°, ranges from 5.2 to 9.1 μN for scratching angle 45°, and ranges from 1.5 to 2.4 μN for scratching angle 90°. When the feed is 50 nm, the normal load for ripple formation ranges from 16.4 to 32.8 μN for scratching angle 0°, ranges from 17 to 25.2 μN for scratching angle 45°, and ranges from 13.7 to 22 μN for scratching angle 90°. By analyzing the obtained results, it also can be found that the scratching direction has a considerable effect on the machining parameters for ripple formation. For the three scratching angles investigated, the value and range of the normal load all increased with feed. In contrast, the value of the normal load for ripple pattern formation under the three scratching angles are ranked as 0° > 45° > 90°.

Figure 3b,c,d shows the relationships between scratching parameters and the periods of the ripples. For feeds from 20 to 40 nm, the range of the normal load changes from 6.4 μN to 21 μN, 5.2 μN to 15 μN, and 1.5 μN to 14 μN for scratching angles of 0°, 45°, and 90°, respectively. Meanwhile, the period changes from 250 nm to 580 nm, 270 nm to 450 nm, and 230 nm to 500 nm for scratching angles of 0°, 45°, and 90°, respectively. For different scratching directions, the tip scratch face, the scratch edge, and the cantilever deformation are all different. The tip scratch face and the scratch edge affect the contact area, and the cantilever deformation affects the actual normal load acting on the sample surface in scratching test, which has been discussed in detail in our previous work [17]. The contact area and the actual normal force will directly affect the contact press, which is the important factor for forming the ripple structures [15]. For the three scratching angle, the contact area is the same due to the scan-scratch trace. So, the tip edge and faces have no effects on the different scratching angles. But, the actual normal load follows the order 0° < 45° < 90°, which means that in order to get the same contact press, the normal load follows the order 0° > 45° > 90°. For the change of the period scope in different scratching directions, it may be due to the change of the actual normal load under each scan-scratching direction. Therefore, for the three scratching angles, the normal load for ripple formation follows the order 0° > 45° > 90°, and the period scope for the ripples formed is 0° > 90° > 45°.

### 3D complex nanodot array formation based on ripples formed with different scanning angles

**Figure 4:**
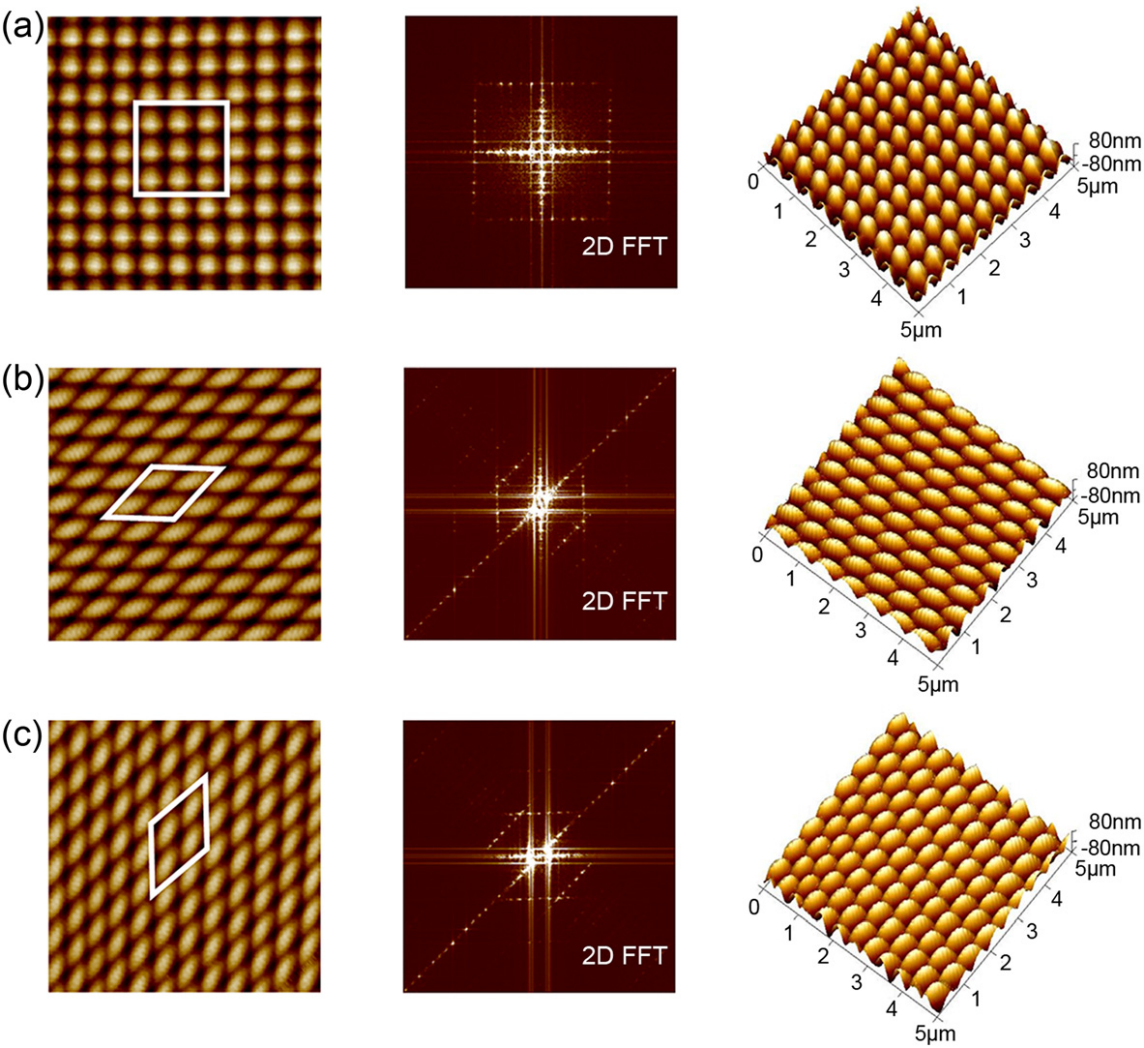
**Morphologies and 2D FFT images of 3D nanodot arrays.** The scratching angles **(a)** 90° and 0°, **(b)** 90° and 45°, **(c)** and 0° and 45° of the two-step scratching method.

Based on the above results, the orientation and period of ripples can be controlled by modifying the scratching angle, feed, and normal load. We then used our two-step scratching method (as shown in Figure 1c,d) to fabricate 3D nanodot arrays on PC surfaces.Firstly, to fabricate nanodots with a size of 500 nm, we chose two-step scratching traces (as shown in Figure 1c) using scratching angles of 90° and 0° for ripple formation with a period of 500 nm. We used a feed of 40 nm and normal load of 14 μN for a scratching angle of 90° and a normal load of 17.3 μN for a scratching angle of 0°. The morphology and fast Fourier transform (FFT) image of the obtained pattern are shown in Figure 4a. The nanodots are arranged with high periodicity in both horizontal and vertical directions. Secondly, we used scratching angles of 90° and 45° (as shown in Figure 1d) to form ripples with a period of 450 nm. A feed of 40 nm and normal load of 11.8 μN were used for a scratching angle of 90°, and load of 14.8 μN was used for a scratching angle of 45°. The morphology and FFT image of the resulting pattern are illustrated in Figure 4b. The formed dots are similar to diamond-shaped nanodots with a length of 550 nm, which was derived from geometric relations. Because the ripples are all oriented perpendicular to the scratching direction, the sides of the obtained diamond dots are parallel to and with an angle of 135° to the horizontal line (highlighted by the white area in Figure 4b). Finally, we used scratching angles of 0° and 45° (as shown in Figure 1e) to scratch the PC surface. Using a feed of 40 nm and normal load of 15.8 μN for a scratching of angle 0° and load of 14.8 μN for a scratching angle of 45°, we formed ripples with a period of 450 nm. The morphology and a FFT image of the fabricated surface are presented in Figure 4c. The length and shape of the dots are the same as the diamond-shaped nanodots above, except that the orientation of the dots has changed, with the sides perpendicular to and with an angle of 135° to the horizontal line (indicated by the white area in Figure 4c).

The above experimental results reveal that the length and orientation of nanodots can be regulated by manipulating the period of the ripples for a selected scratching direction. Using our two-step scratching method, by changing the period of the ripples formed using different scratching angles, complex, controllable 3D nanodot arrays can be fabricated easily.

### Mechanism of ripples formation

**Figure 5:**
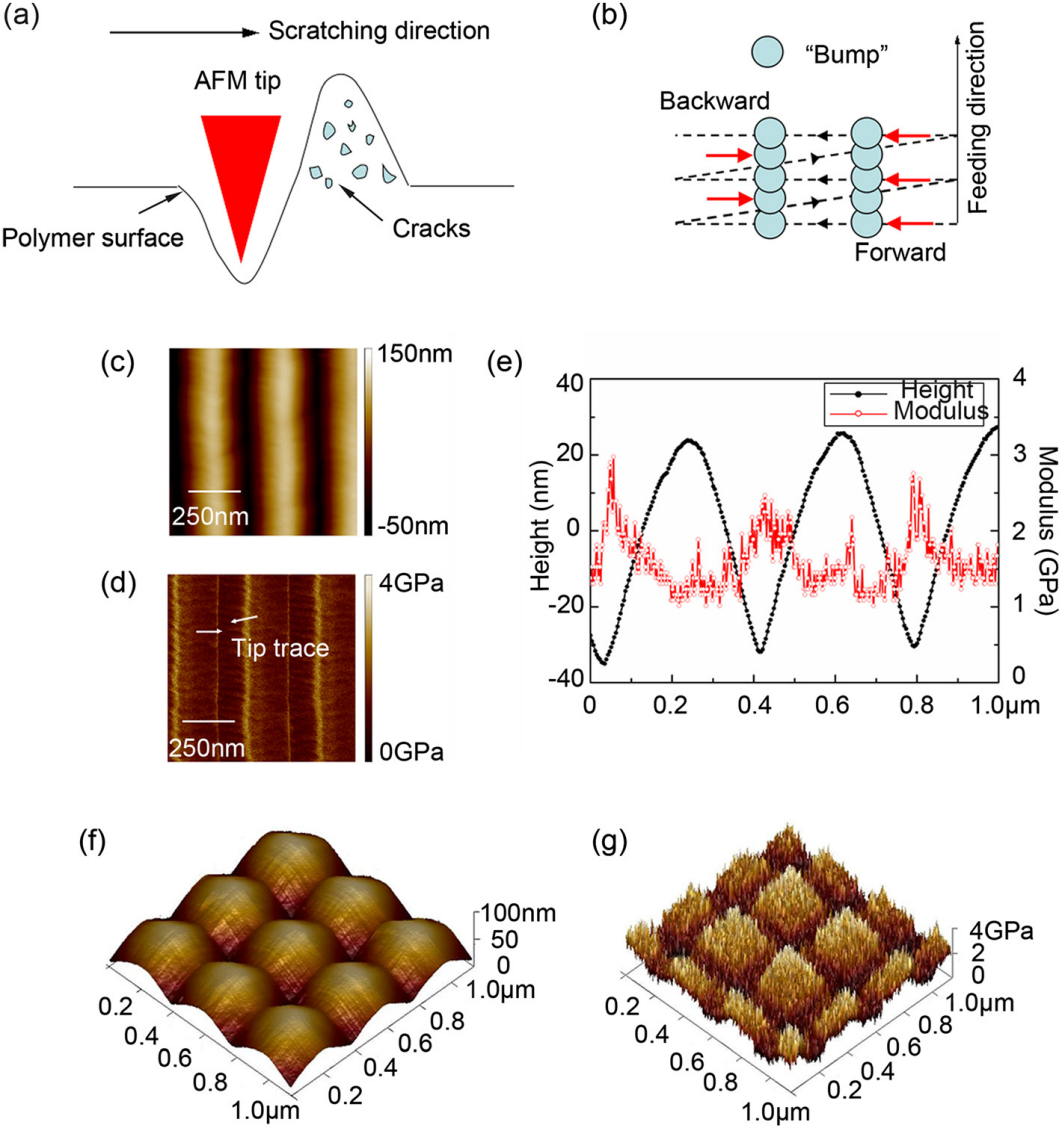
**Schematic of the ripple formation mechanisms by an AFM tip. (a)** Schematic of the bump formation with many cracks and **(b)** the cartoon model for the ripple formation. **(c)** AFM morphology, **(d)** modulus image, and **(e)** cross-sections of a ripple structure. **(f)** The topography and **(g)** modulus image of a 3D nanodots structure.

As shown in Figure 5a,b, the process of ripple formation on PC sample surface can be presumed as an interaction of stick-slip [11] and crack formation [12] processes. When the tip scratches along the fast scanning direction, the AFM tip indents the polymer surface and starts to push the surface material. In practice, the tip still sticks to the surface and is forced to hop over until the polymers that builds up in front of the tip offers enough resistance, so the bump is formed. Because the movement of the tip is a zigzag trace, the formed bump will be pushed forward and backward, and the rippling structures perpendicular to the scratching direction can be fabricated. For the typical ripple structures, the AFM morphology and modulus images are shown in Figure 5c,d. It can be found that the tip trace is clearly at the grooves but blurry at the ridges, which also confirmed that such ripples structures could be a stick-slip phenomenon. The cross-sections of the height and Young’s modulus of the ripples are shown in Figure 5e. The moduli are about 1.5 and 2.5 GPa at the ridges and grooves, respectively. For the raw PC surface, the modulus is about 2.45 GPa. The changing of the modulus may be a consequence of the crack existing within the bumps, which agrees well with the model that proposed by Dr. Khrushudov [12], as shown in Figure 5a. For the 3D nanodots arrays, the AFM morphology and modulus images are shown in Figure 5f,g. The modulus images demonstrate that the whole structures have different mechanical response. The bumps have a low modulus and the hollows have a high modulus, which also could be attributed to the tip-induced cracks formation. Therefore, the mechanism for the occurrence of such rippling structures can be presumed as an interaction of stick-slip and crack formation processes.

## Conclusions

Directional ripple patterns with perfect periodicity can be formed on PC surfaces by scratching zigzag patterns with an AFM tip. The range of normal load and feed used for ripple formation can be obtained to modulate the period of the ripples. By combining scratching angles of 90° and 0°, 90° and 45°, and 0° and 45° in two-step machining, we fabricated nanoscale dot and diamond-dot structures with controlled size and orientation. The typical rippling of the polymer surface can be presumed as a stick-slip and crack formation process. This study reveals that AFM-based nanomachining can be used to fabricate controllable complex 3D nanoripples and nanodot arrays on PC surfaces.

## Abbreviations

AFM: atomic force microscope; PC: polycarbonate; 3D: three-dimensional.

## Competing interests

The authors declare that they have no competing interests.

## Authors’ contributions

YDY and YS carried out the design and drafted the manuscript. JRL participated in the experiments. ZJH and XSZ assisted with the optimization and proofed the manuscript. All authors read and approved the final manuscript.
